# Supplementary material for: Developing a socio-ecological model for community engagement in a health programme in an underserved urban area
Source: PLoS One. 2022 Sep 26;17(9):e0275092. doi: 10.1371/journal.pone.0275092 (PMC9512167; doi:10.1371/journal.pone.0275092)
Supplement: S1 Table — (DOCX) [file pone.0275092.s001.docx]

# Supporting Information

# S1 Table. Workshop guide for Support Standard

| **Item** | **Timings** |
| --- | --- |
| **1. Welcome and share Agenda** | 5 minutes |
| **2. Present Planning activities from last week and ask for further feedback/comments** | 10 minutes |
| **3. Review of the Scottish National Standards – Support** | 10 minutes |
| **4. Breakout session 1**)  **Focus of session is Support:**  *We will identify and overcome any barriers to participation.*  [Examples of support needs are: suitable transport, caring for dependents (e.g. childcare), personal care, access to interpreters, communication aids, meetings at appropriate times, access to online resources/communication methods, out-of-pocket expenses]  Prompts and questions:   1. How have we/can we assess the support needs of our communities (to include everyone)? | 10 minutes |
| **5. Feedback on breakout group** **1)** Share with full meeting | 15 minutes |
| **6.** **Breakout session 2)**  **Support:** Prompts and questions   1. What are the practical barriers which make it difficult for people to take part in engagement activities and how can we remove these barriers? 2. How can we provide independent support for groups involved in the engagement process? | 20 minutes |
| **7. Feedback on breakout group** **2)** Share with full meeting | 15 minutes |
| **8. Next steps: To populate and refine Support data collected today and present at next session / close meeting** | 5 minutes |
