# Supplementary material for: Developing a socio-ecological model for community engagement in a health programme in an underserved urban area
Source: PLoS One. 2022 Sep 26;17(9):e0275092. doi: 10.1371/journal.pone.0275092 (PMC9512167; doi:10.1371/journal.pone.0275092)
Supplement: S2 Table — (DOCX) [file pone.0275092.s002.docx]

**S2 Table. Full list of influences on community engagement discussed by workshop participants and their socioecological environments**
