# Supplementary material for: Developing a socio-ecological model for community engagement in a health programme in an underserved urban area
Source: PLoS One. 2022 Sep 26;17(9):e0275092. doi: 10.1371/journal.pone.0275092 (PMC9512167; doi:10.1371/journal.pone.0275092)
Supplement: Attachment — Submitted filename: PONE-D-21-26119.docx [file pone.0275092.s003.docx]

Remarks to the Author:

*It is important to note that I am an expert in network science for complex social systems and interest in social mobility and gathering. However, I’m not an expert on sociology.

A. **Originality and the key results**

As far as I know, it seems to be the first application of socio-ecological analysis to community engagement in an ethnically diverse and deprived urban area. The author introduces the socio-ecological model with multiple layers of influence on community engagement activities (CEA) in six environmental factors: individual, political, physical, technological, economic, and socio-cultural. Each factor is comprehensively analyzed and presented with its associated influence as well as its own influence on community engagement.

The author highlights the importance of the overall influences of the socio-cultural environment which include social support, trust, relationships in communities, and ethnicity or language. At the same time, the author presents multi-factored aspects needed to ensure community engagement activities such as time spent on CEA, transparency through democratic process, and access to technology or communal spaces.

Finally, the author suggests that considering multiple environmental influences and incorporation of the socio-cultural environment can be more effectively developed the community engagement activities.

B. **Data and model coverage**

The co-production process that has been analyzed in this manuscript only represents the communities within three electoral wards that are considered deprived and have diverse ethnic populations. Also, workshops have some limitations in the number of community members or the existence of under or unrepresented groups as mentioned in the manuscript.

Due to the specificity of these data, further detailed explanations of the ethnic, economic, and cultural backgrounds of the restricted areas or participated communities are needed to avoid the error of hasty generalization and to specify the scope of application of the model.

C. **Suggested improvements**

The framework analysis and developed matrix structure of key socio-ecological themes are adopted to analyze the data. Although framework analysis lists environments that influence community engagement, the evidence in the results is insufficiently descriptive so statistical analysis or regression methods are recommended to support the results.
